# Supplementary material for: Patterns of Intron Gain and Loss in Fungi
Source: PLoS Biol. 2004 Nov 30;2(12):e422. doi: 10.1371/journal.pbio.0020422 (PMC532390; doi:10.1371/journal.pbio.0020422)
Supplement: Table S1 — Also available at http://genes.mit.edu/NielsenEtAl/. (4.3 MB ZIP). [file pbio.0020422.st001.zip › NielsenEtAl/html/1336.html]

AN4217.1.NCU03761.1.MG06950.1.FG07162.1


```
 CLUSTAL W (1.82) Multiple Sequence Alignments - Introns Inserted


Sequence 1: NCU03761.1	404 aa
Sequence 2: MG06950.1	344 aa
Sequence 3: FG07162.1	341 aa
Sequence 4: AN4217.1	425 aa
Alignment Length: 475 aa
Number Identitical Residues: 94 aa
Alignment Score (without introns) 5689


MG06950.1 	MGSY----SVEPVKPWVETPCLYSPQVSRAAGC2NIYLKLDNLQPSGSFKSR~GIGNMMQ
NCU03761.1	MGSVSFPASSEPPKPWVETPLIYSAQLSNAAGC~NIYLKLENVQPSGSFKSR2GIGNMML
FG07162.1 	MGSL----DSGAKLPWIKTPCIISTQLSRLTGC2NVYLKLENLQPSGSFKTR~SISSLMT
AN4217.1  	MAFV-------KRIPWIETPLIESASLSKTAGC2RVFLKLDLLQPSGSFKSR2GIGNLIC
          	*.            **::** : *..:*. :** .::***: :*******:* .*..:: 

MG06950.1 	RAIALTP-------------------RDDVHFYCPSGGNAGLACATSAASLGKPATIVVP
NCU03761.1	RSTLPSPPSSPSSSSTTSFQEPLTTNQRKIHFFCSSAGNAGLACATTAHTLACPATIVVP
FG07162.1 	QAAAAAT--------------------GPVHFYCSSGGNAGLACATSAISLGHKATIVMP
AN4217.1  	NALQDPAN-----------------RGKELHFFSSSGGNAGLAAVIAARDLGCRCTVVVP
          	.:   ..                      :**:..*.******.. :*  *.  .*:*:*

MG06950.1 	IKTPAHMVEKLEALGA-EVLRRGGSIAEADAYLKSQLLIN--------------------
NCU03761.1	TSAQEPVLRRLRGLGA-TVVIHGSSWFEADTHLREVVMPASALEDLQESSSGGEEGEKTK
FG07162.1 	TIVSPLMKSKLLDLGV-EVHQKGKNWAECDKYMREELVAN--------------------
AN4217.1  	YSTKPMMITKLREAGATDVIQHGDSWFEADTYLRETFIENQ------------DQGGDAA
          	  .   :  :*   *.: *  :* .  *.* :::. .:  .            ... .: 

MG06950.1 	DPNGVYVSPFDHPDIWEGASSIVDEITTQMRL--QPGGPKEADGIVCNVGGGGLLIGIMD
NCU03761.1	QTKQVYVPPFDHEDIWSGAATLMDEVAEQMRV--VHR--AYVDAVVCNVGGGGLLNGIME
FG07162.1 	DPSAIYVAPFDDPRLWDGAATMVDEIRDQL--------EDRVDGIVCSVGGGSLVNGLMQ
AN4217.1  	TKRNIYVPPFDHPQIWKGVGTMIDEIAQQMPPRDVSKGSFPADAVICSVGGGGLFNGVVE
          	    :**.***.  :*.*..:::**:  *:   .   .   .*.::*.****.*. *:::

MG06950.1 	GLDRIYCGRPQ-------------NQPRVLAVETRGAESLDASIRAGELVTLPNITSICS
NCU03761.1	GVWGPSRNPGTGKEGGLGKEVGGGKEPRVLAIETVGADSFNQSVKAGELVKMKQITSIAS
FG07162.1 	GIESRPWLG---------------TKPAVLAVETEGADSLNASVLAGEHVTLPEMTSIAT
AN4217.1  	GLGRHLATKNGDAPG---------GKVRVLAVETEGTDSLALSLRKGTLQPLPAITSLAT
          	*:         .  .          :  ***:** *::*:  *:  *    :  :**:.:

MG06950.1 	SLGVARVAPRAYEWTKA-AAG-QLATTVVDDADAVMGSVKFLDDARLLVEVACGATISVV
NCU03761.1	SLGAVQVSKKTWEWSQM-VGGENLVSATVTDAEAAMASVRFADEHRMLVEVSCGATLATA
FG07162.1 	SLGATRVSEQTWKWSQH-PSG-MMKSLVVSDADAAISTVRFADDALQLVEVACGAALAIA
AN4217.1  	SLGALQVAPQTLKNAQCPPARVDVVSVVGSDAEAAQGVIRLADEHRLQVELACGISVEVG
          	***. :*: :: : :: . .   : : .  **:*. . ::: *:    **::** ::   

MG06950.1 	YNGQLRQKLGKGLSDAEWAEKNIVLVVCGGSHTTLEALLRYREEFGV------~------
NCU03761.1	YNGDLRRYLGKGLSDEEWRNVNVVMVVCGGSHVSVDVLKAYREKYGP--EIAW~A-----
FG07162.1 	YRGDLRDVLGKGLSDEEWSKTNVVFVVCGGSGITLSMLSEYREKYSG--QSSI~KV----
AN4217.1  	GSVKLRDYMPDLTPDS-----RIVIVICGGSNVTAEMVVEYRKRLHDGWDSAY2AAFASP
          	   .**  : .  .*      .:*:*:****  : . :  **:.   . . :     ::.

MG06950.1 	-----------------------~--------~---------------------------
NCU03761.1	-----------------------~--------~---------------------------
FG07162.1 	-----------------------~--------~---------------------------
AN4217.1  	AVGSVVHAFDAILGSQVPVLFLM1YPSICSSC1KAASINKKSTMHSSFTWRSRQIQTSTV
          	: .:   : .:  .:. .       .: .::. .::: ...::  :: :  : . .::: 

NCU03761.1	
MG06950.1 	
FG07162.1 	
AN4217.1  	
          	
```
